# The role of the gut microbiome and microbial metabolism in mediating opioid-induced changes in the epigenome

**DOI:** 10.3389/fmicb.2023.1233194

**Published:** 2023-08-21

**Authors:** Udhghatri Kolli, Sabita Roy

**Affiliations:** Department of Surgery, University of Miami Miller School of Medicine, Miami, FL, United States

**Keywords:** opioids, epigenetic, microbiome, microbial metabolism, metabolites

## Abstract

The current opioid pandemic is a major public health crisis in the United States, affecting millions of people and imposing significant health and socioeconomic burdens. Preclinical and clinical research over the past few decades has delineated certain molecular mechanisms and identified various genetic, epigenetic, and environmental factors responsible for the pathophysiology and comorbidities associated with opioid use. Opioid use-induced epigenetic modifications have been identified as one of the important factors that mediate genetic changes in brain regions that control reward and drug-seeking behavior and are also implicated in the development of tolerance. Recently, it has been shown that opioid use results in microbial dysbiosis, leading to gut barrier disruption, which drives systemic inflammation, impacting the perception of pain, the development of analgesic tolerance, and behavioral outcomes. In this review, we highlight the potential role of microbiota and microbial metabolites in mediating the epigenetic modifications induced by opioid use.

## 1. Introduction

Opioids are highly effective analgesics for the treatment of perioperative and postoperative pain. However, their use is limited by the development of analgesic tolerance, dependence, opioid use disorders, addiction, and comorbidities associated with opioid use. The present opioid crisis is multifactorial, arising from increased use, misuse, and abuse of prescription opioids as well as the recreational use of opioids. The rate of opioid overdoses, mainly involving synthetic opioids such as fentanyl, is on the rise, and in the year 2020, ~68,630 overdose deaths involving opioids (CDC WONDER, 2021). Concerning opioid overdoses, the overall economic burden of opioid use disorder and fatal overdose deaths is estimated at USD$ 1,072 billion, including healthcare and addiction recovery costs (USD$ 35 billion), costs related to criminal justice ((USD$ 14.8 billion), lost quality of life (USD$ 390 billion), and lost productivity ($549 billion) to opioid overdose (Florence et al., 2021). Despite the staggering effect of opioid use disorder on public health and socioeconomic status, few effective treatments exist for delaying the development of tolerance and combating addiction.

Opioid use adversely impacts gastrointestinal (GI) homeostasis by impairing gut motility, barrier function, and microbial composition. Opioid use-associated gut microbial dysbiosis (defined as alterations in microbial composition and metabolism) is emerging as a key factor in modulating opioid function and comorbidities associated with opioid use (Wang and Roy, 2017; Akbarali and Dewey, 2019; Sharma et al., 2019; Zhang et al., 2019; Meng et al., 2020; Ren and Lotfipour, 2020; Eitan et al., 2021). The gut microbiome and its metabolites are known to alter the epigenetic landscape in various disease states or conditions such as cancer, diabetes, obesity, autism spectrum disorders, aging, and chronic kidney disease (Lam et al., 2016; Krautkramer et al., 2017a,b; Pan X. et al., 2018; Sobhani et al., 2019; Cappuccilli et al., 2020). One of the mechanisms by which the microbiota impacts the host's physiology is through the production of bioactive compounds and metabolites that affect the epigenetic machinery and modulate their activity (Lynch and Pedersen, 2016; Piazzi et al., 2019).

An increasing number of studies in the past two decades have highlighted the role of epigenetic modifications in substance use disorders, which is summarized in Table 1. In this study, we review the current literature on epigenetic changes (DNA methylation and histone modifications) associated with substance use disorders, focusing on opioids. We aim to highlight the role of microbial metabolism and metabolites as mediators of epigenomic changes by focusing on choline, methionine, s-adenosyl methionine (SAM), folate, and short-chain fatty acids (SCFAs) in modulating epigenetic changes.

**Table 1:** Summary of epigenetic changes observed with substance use.

**Substance of abuse**	**Treatment/model**	**Epigenetic mark**	**Tissue**	**Gene targets**	**Behavior response**	**Manipulation**	**Manipulation-behavior outcome**	**References**
Heroin, opioid addicts, abusers, prescription opioids	Human	↑ DNA methylation- OPRM1 promoter, LINE-1	Plasma, blood, saliva, brain	–	Corelated with pain and inadequate pain relief	–	–	Nielsen et al., 2009; Chorbov et al., 2011; Viet et al., 2017; Xu et al., 2018; Sandoval-Sierra et al., 2020
Morphine	*In vitro*	↑ 5mc levels at CpG islands, gene bodies	Neuronal stem cells	TET1 activity	Neuronal stem cell proliferation	–	–	Liang et al., 2020
Heroin and methylamphetamine	Human	↓ DNA methylation-BDNF promoter	Blood	BDNF	Addiction behavior			Xu et al., 2016
Morphine	Rats, inflammatory pain model	↑H3acetylation	Dorsal root ganglia astrocytes	–	–	-		Li et al., 2012
Heroin	Rats, self-administration model	↓Histone acetylation-Debrin promoter	Nucleus acumens	F-actin	Heroin induced plasticity	Debrin KO and HDAC2 inhibition	Rescued drug seeking and relapse behavior	Martin et al., 2019
Morphine, cocaine	Mouse	↑SIRT2	Nucleus acumens	–	Enhancement of morphine reward	–	–	Ferguson et al., 2013
Morphine	Mouse	↑H3K9ace- BDNF, pdyn promoters	Spinal cord, dorsal root neurons	BDNF, pydn	Opioid induced hyperalgesia	HDAC inhibition-SAHA	Prolonged opioid induced hyperalgesia	Liang et al., 2014
Morphine	Mouse	–	Prefrontal cortex	CaMKIIα	Morphine tolerance and dependence	HAT inhibition-Curcumin	Delayed morphine tolerance and dependence	Hu et al., 2015
Morphine	Mouse	–	Brain	BDNF	Morphine tolerance	HAT inhibition-Curcumin	Attenuated morphine tolerance	Matsushita and Ueda, 2009
Morphine	Mouse	–	Spinal cord	CaMKII	Opioid induced hyperalgesia	HAT inhibition-Curcumin	Attenuated opioid induced hyperalgesia	Hu et al., 2016
Morphine	Mouse	↑Histone acetylation-BDNF promoter	Ventra medial prefrontal cortex	BDNF	Conditioned place aversion/withdrawal	HDAC inhibition	Extinction of aversive memory-withdrawal	Wang et al., 2012
Morphine		↑Histone acetylation-CXCL12 promoter	Hippocampal CA1 neurons	CXCL12	Morphine seeking behavior	–	–	Liu et al., 2020
Morphine	Mouse	↑H3K9ace-BDNF	LC		Withdrawal	–	–	Mashayekhi et al., 2012
Morphine	Mouse	↓H3K9me2-FOSB	Nucleus acumens	FOSB	–	–	–	Sun et al., 2012
Morphine	Mouse	–	Basolateral amygdala	BDNF, delta FOSB, CREB activation	–	Pretreatment-HDAC inhibitor-TSA	Facilitated morphine induced CPP	Wang Y. et al., 2014
Morphine	Mouse	↓H3K9me2	Nucleus acumens	G9a	Morphine reward	G9a overexpression	Reduced morphine reward behavior	Sun et al., 2012
Morphine	mouse	↑H3K9ace	Ventral tegmental area-Dopamine neurons	HDAC2		HDAC2 inhibition by CI-994	Glutamargic potentiation	Authement et al., 2016
Morphine	Human/pre-operative use	↑H3K9ace-BDNF, pdyn	Spinal cord	BDNF, pydn	Opioid induced hyperalgesia	–	–	Sahbaie et al., 2016

## 2. Impact of opioid use on gut microbial composition and function

The GI tract is home to trillions of microorganisms (microbes, fungi, viruses, archaea, and bacteriophages). Their combined genetic materials, metabolites, and bioactive compounds are collectively termed the microbiome. Under homeostatic conditions, the host and the microbiome maintain a symbiotic relationship, and the gut microbes are known to impact the host's physiology through the production and modulation of epigenetically active compounds (choline, methionine, SAM, folate, and SCFAs), neurotransmitters, bile acids, vitamins, and nutrients (Dinan and Cryan, 2017; Silva et al., 2020; Eitan et al., 2021).

Gut bacterial dysbiosis, characterized by the expansion of pathogenic and opportunistic pathogens at the expense of beneficial and commensal bacteria, is observed in various models of opioid use, including clinical and preclinical studies utilizing rodents, non-human primates, and zebrafish. While opioid-associated changes in gut bacterial composition varied in different studies, mainly depending on the drug of choice, treatment dosage, administration route, and length of opioid treatment, microbiome analysis conducted in various studies has established that opioid use leads to the expansion of pathogenic bacteria such as *Enterococcus faecalis* and the depletion of probiotic bacteria of the genera *Lactobacillus, Clostridium, and Bifidobacterium* (Meng et al., 2013; Banerjee et al., 2016; Acharya et al., 2017; Kang et al., 2017; Wang et al., 2018; Sharma et al., 2019; Sindberg et al., 2019; Zhang et al., 2019). The bacteria belonging to the genera *Lactobacillus* and *Clostridium*, which are highly implicated in secondary bile acid deconjugation, decrease with morphine treatment, which is associated with decreased bile salt deconjugation, gut barrier dysfunction, and increased inflammation (Banerjee et al., 2016).

Decreased abundance of *Bifidobacterium* and *Lactobacillus* affects the development of morphine tolerance (Kang et al., 2017; Zhang et al., 2019), and the dysbiotic morphine microbiome alone can drive the development of morphine tolerance (Zhang et al., 2019). Opioid use also leads to a decreased SCFA production potential of the microbiome, as evidenced by a decrease in *Lachnospiraceae, Rumninococcaceae, and Muribaculaceae* in humanized mice (Meng et al., 2020). Microbiome analysis of stool samples from patients undergoing addiction treatment confirmed the preclinical data from mouse models and indicated that opioid use is associated with decreased microbial diversity and decreased relative abundance of butyrate-producing bacteria belonging to the genus *Roseburia* and bile acid deconjugating bacteria of the genus *Bilophila* (Gicquelais et al., 2020).

While the role of bacteria in modulating opioid function has been studied at length, the role of other gut residents such as archaea, fungi, viruses, and bacteriophages remains unexplored. Furthermore, the method by which opioids modulate the metabolic function and transcriptome of the microbiome is also not understood. The use of metagenomics and meta-transcriptomics to understand the transcriptional changes in the microbiome may help in the development of therapeutic interventions to limit morphine-induced microbial dysbiosis.

## 3. Gut microbial metabolism and opioid-induced alterations in DNA methylation

DNA methylation is a dynamic epigenetic modification that involves the enzymatic addition of a methyl group from SAM to the 5' position of cytosine in the context of CpG dinucleotides. It is established by the DNA methylases Dnmt3a and Dnmt3b and maintained by Dnmt1 (Solary et al., 2014; Edwards et al., 2017). The ten-eleven translocation family of DNA demethylases (TET1–TET3) can hydrolyze the 5-methylcytosine (5-mc) to 5-hydroxymethylcytosine (5-hmc), ultimately leading to DNA demethylation (Solary et al., 2014; Kai et al., 2016; Noreen et al., 2019). DNA methylation regulates gene expression and genome stability. DNA methylation at gene promoters is commonly associated with gene repression, while DNA methylation at gene bodies potentially affects transcript elongation or splicing (Robertson and Jones, 2000; Greenberg and Bourc'his, 2019). The gut microbiome influences DNA methylation patterns and contributes to intestinal homeostasis during various developmental phases and in adulthood (Yu et al., 2015; Pan W.-H. et al., 2018; Ansari et al., 2020). While microbiome-regulated expression of *Dnmt3a* and *Tet3* has been shown to be involved in the alterations in DNA methylation levels, the role of microbial metabolites has not been assessed, and the role of bacterial metabolites cannot be ignored.

The one-carbon cycle is a metabolic process that supplies one-carbon units for nucleotide synthesis and methylation reactions, including DNA and histone methylation events. The metabolites choline, methionine, and betaine can influence DNA methylation by serving as precursors for the generation of the universal methyl donor, SAM, through one-carbon metabolism (Friso et al., 2017). The gut microbiome could alter choline bioavailability in the host by metabolizing choline to trimethylamine (TMA) (Martinez-del Campo et al., 2015; Rath et al., 2017; Romano et al., 2017). Choline-utilizing gut bacteria compete with the host for choline and induce a choline-deficient phenotype, resulting in decreased DNA methylation in various brain regions and leading to increased anxiety in mice. The behavioral phenotype was observed to be transgenerational, highlighting the role of bacterial choline utilization in creating a lasting effect on the epigenome and resulting in an altered behavioral response (Romano et al., 2017).

**Figure 1:**
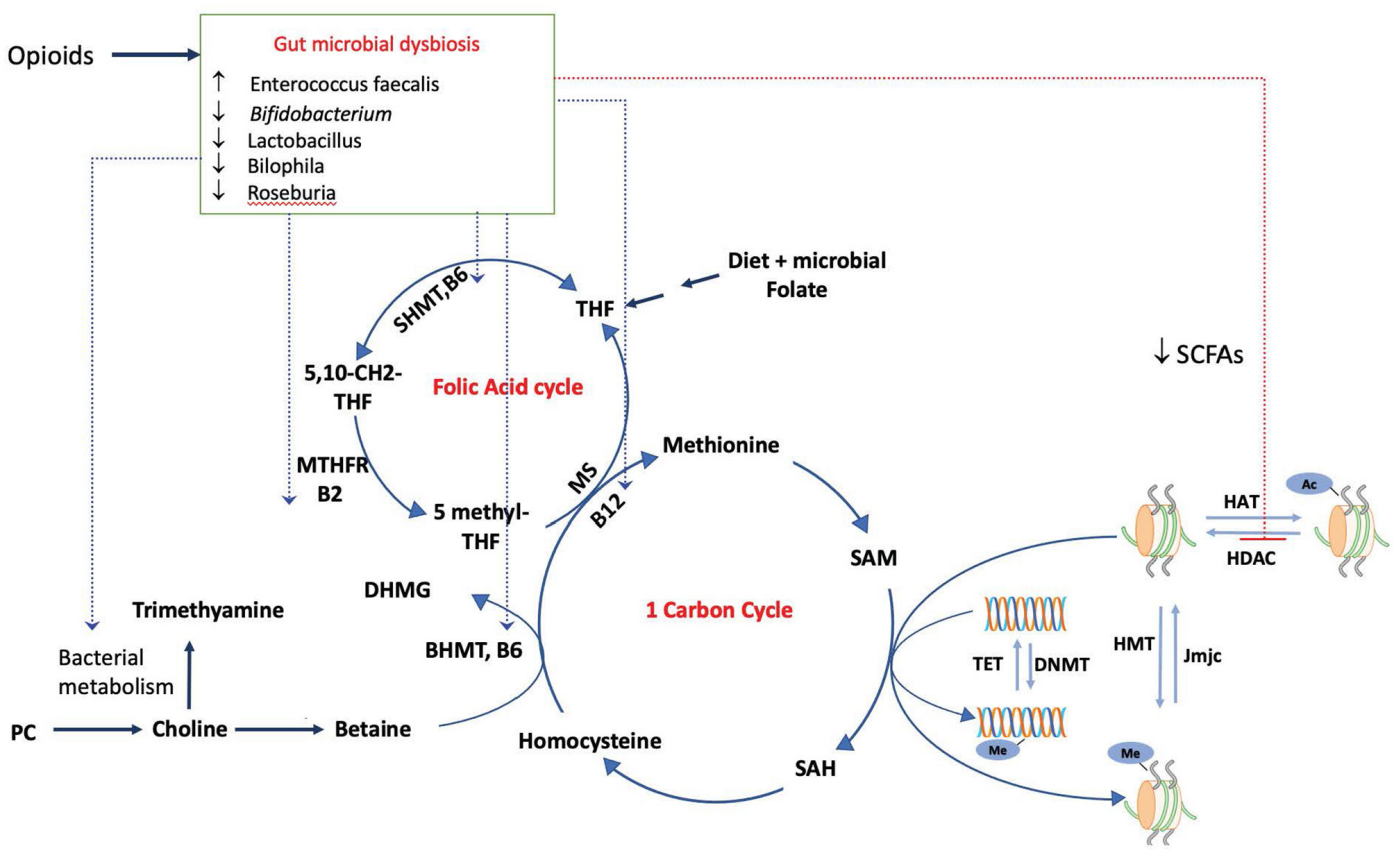
Modulation of the epigenome by the dysbiotic opioid microbiome. Opioid use alters the gut microbial composition and metabolism, impacting the production and availability of SCFAs, which inhibit the activity of HDACs and impact histone acetylation. Morphine microbiome would impact the availability of SAM, a universal methyl donor required by both DNMTs and HMT for methylation of DNA and histones, by modulating the metabolism and availability of metabolites choline, folate, and vitamins (B6, B2, and B12). These serve as cofactors for reactions in one-carbon and folic acid cycles, SAM, and S-adenosylmethionine. SAH, S-adenosyl homocysteine; SHMT, serine hydroxymethyltransferase; MTHFR, methylenetetrahydrofolate reductase; MS, Methionine synthase; DNMT, DNA methyltransferase; HMT, histone methyltransferase.

Folic acid is an important micronutrient, and the folic acid cycle is linked to the one-carbon cycle. The conversion of 5-methyl tetrahydrofolic acid (5-mTHF) to tetrahydrofolic acid (THF) in the folate cycle is coupled with the conversion of homocysteine (hCYS) to methionine, which is subsequently converted to SAM, the universal methyl donor (Figure 1). Thus, folate is important for maintaining SAM levels and for biological methylation reactions, including DNA and histone methylation (LeBlanc et al., 2013; Krautkramer et al., 2016, 2017a,b; Bhat and Kapila, 2017; Gurwara et al., 2019; Kok et al., 2020). The bacterial strains *Bifidobacterium adolescentis* (*MB 227* and *MB 239*) and *Bifidobacterium pseudocatenulatum* (*MB 237*) are high folate producers. Folate production by bacteria is independent of host folate levels (Pompei et al., 2007a,b). *Lactococcus lactis, Lactobacillus plantarum, Streptococcus thermofilus, B. adolescentis, B. pseudocatenulatum, and Bifidobacterium catenulatum* are folate producers in the gut (Kok et al., 2020), indicating that the microbiome plays an important role in maintaining folate levels in the host. Morphine use is associated with decreases in *Bifidobacterium* and *Lactobacillus* Kang et al., 2017; Xu et al., 2017; Hakimian et al., 2019; Zhang et al., 2019), and it could potentially reduce folate levels, thereby impacting DNA and histone methylation.

The B-complex group of vitamins can affect the host epigenome by serving as cofactors for many enzymes in the one-carbon metabolism and the folate cycle. Humans cannot synthesize vitamins belonging to the B-complex group, and these vitamins are thus obtained through diet. Endogenous gut microbiota are producers of riboflavin (vitamin B2), niacin (B3), pantothenate (B5), pyridoxine (B6), folate (B9), and cobalamin (B12) and contribute to the host's vitamin B supply, thus affecting the host epigenome. Probiotic bacteria belonging to the species *Lactobacillus* and *Bifidobacterium* are potent producers of these vitamins (LeBlanc et al., 2013; Krautkramer et al., 2016, 2017a; Bhat and Kapila, 2017; Gurwara et al., 2019). Cobalamin (vitamin B12) affects methylation reactions by serving as a cofactor for the enzyme methionine synthase (MS), which catalyzes the conversion of homocysteine (hCYS) to methionine (Figure 1). Small intestinal bacterial overgrowth affects vitamin B12 host availability by converting cobalamin to host-inaccessible cobalamin corrinoids (Jory, 2019). The method by which morphine-induced expansion of pathogenic bacteria and decreased abundance of *Bifidobacterium* and L*actobacillus* affects the levels of the B-complex group of vitamins has to be determined to fully understand the role of morphine-induced expansion of dysbiotic bacteria in DNA modulation and histone methylation.

Gut microbial colonization has been shown to affect the DNA methylation and expression of inflammation-associated genes in neonates (Cortese et al., 2016), and short-term methionine restriction has been shown to alter global hepatic methylation in adult mice (Mattocks et al., 2017). A recent study indicated that the co-administration of morphine and SAM, a universal methyl donor, resulted in the attenuation of the development of morphine analgesic tolerance as measured by tail flick in rats (Katyal et al., 2017). Collectively, these findings suggest that the microbiome and microbial metabolism can influence the host epigenome by altering the substrates/methyl donors (choline, betaine, methionine, SAM, and SAH) and cofactors (folate, cobalamin, riboflavin, and niacin) of the one-carbon metabolism and the folate cycle (Friso et al., 2017).

The activity of the TET family of enzymes is dependent on oxoglutarate, and the replacement of oxoglutarate with 2-hydroxyglutarate (2HG) results in inhibition of TET enzymes (Han et al., 2018; Knorr et al., 2018; Ternes et al., 2020). A recent study by Knorr et al. showed that the microbial conversion of lysine to succinate involves the production of 2-HG as an intermediate. This result suggests that the microbiome can alter the DNA methylation landscape of the cells, leading to a more hypermethylated state by inhibiting TET enzymes through the production of 2HG (Knorr et al., 2018; Ternes et al., 2020). Interestingly, morphine and naloxone were shown to bind to TET1, inhibit its demethylation activity, and thus facilitate neural stem cell proliferation (Liang et al., 2020). In line with these findings are studies that showed that morphine and the morphine microbiome led to hypermethylated DNA in a cohort of opioid-treated pain patients. These studies showed an increase in methylation at long DNA-interspersed nucleotide elements (LINE-1), which significantly correlated with pain (Doehring et al., 2013). Higher methylation levels observed at LINE-1 indicate that opioid use may lead to global DNA methylation. Additionally, morphine treatment was shown to decrease the redox potential and DNA methylation by inhibiting cysteine uptake through excitatory amino acid transporter type 3 (Trivedi et al., 2014).

### 3.1. Opioid-induced alterations in DNA methylation at the OPRM1 gene and the potential role of the microbiome

Mu-opioid receptor (MOR), encoded by the OPRM1 gene, is a receptor for morphine and morphine derivatives and plays an important role in mediating morphine's analgesic properties and the development of tolerance. Considering the crucial role of μ-opioid receptor signaling, a large number of studies have focused on studying opioid-induced epigenetic modifications that affect the *OPRM1* gene promoter and its expression in relation to opioid-induced behavioral changes. Opioid use is associated with aberrant DNA methylation at the *OPRM1* promoter region, and ethnicity has been identified as an important player in determining the DNA methylation changes. For example, the DNA methylation of the *OPRM1* promoter was higher in the African American population than in the Hispanic and Caucasian populations (Nielsen et al., 2009). Hypermethylation of the *OPRM1* promoter region was identified in the lymphocytes (−18 and +84 CpG sites) of Caucasian heroin addicts on methadone maintenance (Nielsen et al., 2009), the blood and sperm (5, 9, 10, 11, 18, 23, and 24 CpG sites) of male opioid addicts (Chorbov et al., 2011), the blood (126 CpG) of heroin users, and the blood (−60, −32, +27, and +126+140 CpG) of opioid users in the Chinese Han population (Xu et al., 2018). Even though hypermethylation at the *OPRM1* promoter region was observed in opioid users of various ethnicities, the location and extent of hypermethylation seem to depend on the ethnic background.

Hypermethylation of the *OPRM1* promoter was observed in cancer patients taking opioids for pain management, and the extent of methylation was dose-dependent and associated with the lack of inadequate pain relief (Viet et al., 2017). Hypermethylation of the *OPRM1* promoter region was also observed in the saliva of postoperative patients with short-term clinical opioid use, and the extent of methylation correlated with opioid dosage (Sandoval-Sierra et al., 2020), suggesting that even short-term clinical opioid use results in an epigenetic change. Heroin use has also been associated with differential DNA methylation patterns in neurons isolated from the prefrontal cortex region of the postmortem brains of users who died of a heroin overdose (Kozlenkov et al., 2017). Ethnicity appears to play an important part in determining the extent and location of DNA methylation, and it is important to note that different dietary habits and the concomitant effect of diet on the microbiome among people of different ethnicities may be contributing factors to the changes in DNA methylation.

A single nucleotide polymorphism (SNP) at position +188 of the *OPRM* gene promoter resulted in the introduction of a new CpG site at this location, thereby leading to hypermethylation and downregulation of the *OPRM1* gene in the postmortem brain tissue of chronic opioid addicts compared to opioid addicts who did not harbor the SNP in the *OPRM1* gene (Oertel et al., 2012). The A118G SNP in *OPRM1* was significantly associated with the onset and treatment of opioid addiction (Taqi et al., 2019). In a study by Doehring et al., DNA methylation of the OPRM1 promoter (+126, +159 CpG) was higher in the blood leukocytes of opiate addicts. In a separate cohort of postmortem brain tissue (somatosensory cortex and lateral thalamus), no significant changes in the *OPRM1* gene expression were observed in heroin addicts compared to controls (Doehring et al., 2013). In an opioid cohort of postmortem brain tissue from heroin addicts, DNA methylation of the *OPRM1* promoter region did not significantly correlate with *OPRM1* expression levels (Knothe et al., 2016). These findings imply that the *OPRM1* levels observed in peripheral blood may not be indicative of brain *OPRM1* methylation levels, and may not represent brain OPRM1 transcript levels.

The role of the gut microbiome in modulating these changes is not known and needs to be addressed. However, supplementation with probiotic *Lactobacillus acidophilus* was shown to attenuate abdominal pain by mediating the expression of *OPRM1* and cannabinoid receptors (CB2) in the intestinal epithelial cells in an Nf-kb-dependent mechanism (Rousseaux et al., 2007), implying that gut microbiota plays a role in pain perception by regulating *OPRM1* gene expression. Notably, morphine use has been associated with a decrease in the species *Lactobacillus* in the gut (Meng et al., 2013, 2020; Banerjee et al., 2016; Sharma et al., 2019; Zhang et al., 2019). These results collectively imply that the microbiome plays an important role in modulating morphine-induced hyperalgesia. Experimental evidence employing μ-opioid receptor knock out mice also indicated that the endogenous opioid system sets the tone for the basal composition of the microbiome (Banerjee et al., 2016), indicating bidirectional regulation between the μ-opioid receptor and the microbiome.

These studies collectively indicate that morphine use leads to complex changes in global DNA methylation levels that are in part mediated by the microbiome. Evidence from separate studies shows that the microbiome is an important player in the regulation of DNA methylation; however, direct evidence linking the microbiome to opioid-induced changes in DNA methylation is lacking.

## 4. Opioid-induced histone modifications: the microbiome as a potential modulator

Histone acyl modifications, such as histone acetylation, crotonylation, butyrylation, and lysine methylation, are major post-translational modifications present at the N-terminal tails of histones. The post-translational modifications generate distinct marks on the histones, contributing to the diversity of the histones. These post-translational modifications of histones can affect the DNA packaging into the nucleosomes, thus affecting the organization of chromatin and the chromatin-associated process.

### 4.1. Histone acetylation

Histone acetylation is one of the best-characterized acyl modifications. The acetylation of histones neutralizes the positive charge of the histone tails, thereby weakening the interaction between the negatively charged DNA and positively charged histones and opening the chromatin to the transcriptional transcription. Histone acetylation is therefore often linked to transcriptional activation. Histone acetylation is maintained by the addition of the acetyl group from acetyl-CoA by a group of enzymes termed histone acetyltransferases (HATs), and the acetyl group from the lysine present on histone N-terminal tails is removed by the action of enzymes termed histone deacetylases (HDACs). Class I (HDAC 1, 2, 3, and 8) and class II HDACs (HDAC 5, 7, 6, 9, and 10) are zinc-dependent, and class III HDACs belong to the family of NAD+ dependent sirtuins (Sirt1–7) (Lennartsson and Ekwall, 2009; Bannister and Kouzarides, 2011; MolinaSerrano et al., 2019).

In recent years, it has been shown that microbial fermentation of complex dietary fibers results in the production of SCFAs: butyrate, acetate, and propionate. These SCFAs are known to inhibit the activity of HDACs, thus contributing to the changes in the epigenome (Stilling et al., 2014; Sook Lee et al., 2017; Silva et al., 2020). Microbially derived butyrate is a potent inhibitor of HDACs belonging to classes I and II, and it leads to hyperacetylation of the histones and results in increased gene expression. Approximately 2% of the mammalian genome has butyrate-responsive elements, and gene activation and repression by HDAC are mediated through the binding of transcription factors SP1 and SP2 to the butyrate-responsive genes (Davie, 2003). In a recent study by Yuille et al., cell-free culture supernatants from 79 common commensal bacteria were used to screen for SCFA production capacity and HDAC inhibition potential. The cell-free culture supernatants from three strains: *Megasphaera massiliensis MRx0029, Roseburia intestinalis MRx0071*, and *Bariatricus massiliensis MRx1342*, had the strongest HDAC inhibitory capacity, and metabolite analysis revealed that all three strains are potent butyrate producers. The strain *M. massiliensis MRx0029* had the maximum inhibitory effect and is a producer of butyrate and valeric acid, identifying valeric acid as one of the potential HDAC inhibitors of microbial origin (Yuille et al., 2018). Furthermore, *M. massiliensis MRx0029* was shown to inhibit the secretion of IL-6 *in vitro* via the production of SCFAs (Ahmed et al., 2019).

The epigenome, particularly the acetylation and methylation status of histones H3 and H4 in the colon, liver, and adipose tissue, is altered by the microbiome through the production of SCFAs and other bacterial metabolites in a diet-dependent manner. Conventionally raised mice were found to have higher levels of histone acetylation and methylation than germ-free mice, and microbiota transfer increased the acetylation and methylation levels of histones in germ-free mice (Krautkramer et al., 2016). Additionally, the microbiome was shown to regulate microglial properties, functions, and colonization by creating an open chromatin state through the production of SCFAs (Thion et al., 2018). These results clearly indicate that the gut microbiome can alter the epigenome of the host through the production of SCFAs and other metabolites necessary for methylation reactions. A recent study conducted by us showed that opioid use in an HIV model of humanized mice leads to exacerbated microbial dysbiosis, gut barrier damage, and impaired intestinal renewal through HDAC1-mediated suppression of NOTCH signaling. We also showed that morphine treatment leads to decreased HDAC inhibition capacity of luminal content, and pharmaceutical inhibition of HDAC by suberoylanilide hydroxamic acid blocked morphine's suppression of NOTCH signaling and stem cell proliferation (Meng et al., 2020). In another previous study, we showed that morphine treatment inhibits IL-2 production in T-cells by inhibiting acetylation trimethylation of histones and decreasing DNA demethylation at IL-2 promoter regions, thus affecting the T-cell response and increasing susceptibility to infections (Wang et al., 2007). The role of the gut microbiome was not investigated in the latter studies; however, since Interleukin-2 (IL-2) has multiple, sometimes opposing, functions during an inflammatory response, it is plausible that opioid-induced dysbiosis that results in sustained inflammation may drive IL-2 expression, thus contributing to both the induction and the termination of inflammatory immune responses.

### 4.2. Role of histone acetylation in mediating morphine's analgesic effect, tolerance dependence, and hyperalgesia (OIH)

Opioid use is associated with increased histone H3 acetylation in the spinal cord and the development of OIH. HAT inhibition by curcumin attenuated the development of OIH, tolerance, and dependence, whereas HDAC inhibition had the opposite effect and increased side effects, implicating increased histone acetylation in the development of OIH, tolerance, and dependence (Liang et al., 2014). Increased acetylation of lysine at position 9 in histone H3 tail (H3K9 acetylation) at the promoters of BDNF and *pdyn* (prodynorphin) was observed with morphine treatment in the dorsal horn neurons of the spinal cord, contributing to the development of OIH; treatment with the HDAC inhibitor SAHA led to prolonged OIH (Liang et al., 2014). Additionally, early-stage inflammatory pain was associated with decreased acetylation of histone H3 in dorsal root ganglia neurons, and the use of morphine in the mouse model of inflammatory pain resulted in the recovery of histone H3 acetylation in glial cells and neurons, along with increased H3 acetylation in astrocytes (Li et al., 2012), indicating the role of acetylation in mediating inflammatory pain. Preoperative chronic morphine use was associated with increased OIH and reduced analgesic tolerance mediated through increased H3K9 acetylation at BDNF and *pdyn* and concomitant increased expression of these genes in the spinal cord, thus limiting the clinical use of morphine for surgical pain (Sahbaie et al., 2016).

CaMKII is a critical mediator of opioid-induced hyperalgesia (OIH), neuropathic pain (Chen et al., 2009, 2010), and inflammatory pain (Luo et al., 2008). Increased CaMKII expression has been observed in the spinal cord during OIH and is required for both its initiation and maintenance (Chen et al., 2009, 2010). HAT inhibition by curcumin was shown to delay morphine tolerance and dependence by attenuating the activity of CaMKIIα in the prefrontal cortex (Hu et al., 2015) and by attenuating OIH by inhibiting the activity of CaMKIIα in the spinal cord (Hu et al., 2016). In addition to inhibiting CaMKII activity, curcumin treatment was also shown to block morphine-induced upregulation of BDNF and the development of tolerance (Matsushita and Ueda, 2009). In a rat model of bone cancer pain, tumor cell inoculation resulted in the downregulation of OPRM1, which correlated with increased HDAC expression. Furthermore, HDAC inhibition was shown to attenuate morphine tolerance and restore MOR expression, highlighting the role of histone acetylation in the development of morphine tolerance during the progression of bone cancer pain (He et al., 2018).

These results highlight the role of histone acetylation in the development of morphine analgesic tolerance, dependence, and OIH. Pharmacological HDAC inhibition or modulation of the microbiome aimed at the production of bioactive compounds responsible for altering histone acetylation may help overcome the development of tolerance, dependence, and OIH and improve the clinical use of opioids for pain management.

### 4.3. Role of histone acetylation in mediating morphine's rewarding behavior and addiction

Opioid use is associated with alterations in histone acetylation in the brain reward systems, contributing to reward-related learning, memory, drug-seeking behaviors, and addiction. Accumulating evidence suggests that histone acetylation plays an important role in mediating morphine's rewarding behavior in mouse models of morphine-induced conditioned place preference (CPP). For example, pretreatment with HDAC inhibitor trichostatin A (TSA) was shown to facilitate memory formation during the acquisition and extinction of morphine-induced CPP in the basolateral amygdala through increased H3K14 acetylation and a concomitant increase in BDNF, Delta FOSB, and CREB activation (Wang Z. et al., 2014). A similar result of induced CPP was observed with HDAC inhibition by sodium butyrate in the striatum (Sanchis-Segura et al., 2009). Increased histone acetylation of the *Dlg4* gene (disk large homolog 4) encoding for postsynaptic density protein (PSD-95) mediated by pCREB in the ventral tegmental area (VTA) contributed to morphine-induced CPP and rewarding behavior (Wang Y. et al., 2014). Additionally, increased histone acetylation was observed at the promoter of BDNF in the ventral medial prefrontal cortex following conditioned place aversion (CPA), and HDAC treatment facilitated the extinction of aversive memory associated with morphine withdrawal (Wang et al., 2012).

Increased acetylation has also been implicated in morphine-induced neuroinflammation, as increased acetylation and upregulation of *cxcl12* were observed in hippocampal CA1 neurons, driving morphine-seeking behavior (Liu et al., 2020). Morphine use was also associated with synaptic abnormalities mediated through increased HDAC2 expression. HDAC2-mediated decrease in H3K9 acetylation was observed in ventral tegmental area (VTA) dopamine neurons and is reversible by HDAC2 inhibition with the drug CI-994 (Authement et al., 2016).

Depletion of the microbiome by prolonged antibiotic treatment resulted in decreased SCFAs and increased cocaine-mediated CPP, while supplementation with SCFAs resulted in a behavioral phenotype similar to that of regular mice, implying that microbial SCFAs are responsible for cocaine-seeking behavior (Kiraly et al., 2016). However, the results observed with cocaine cannot be directly extrapolated to other substances of abuse. The vast wealth of data obtained through studies aimed at pharmaceutical manipulation of HAT and HDAC implies that histone acetylation plays an important role in mediating morphine's rewarding behavior. While these studies established that aberrant epigenetic modifications at candidate genes, such as *OPRM1, BDNF, PDYN, CaMKII*, and *CXCL12*, and pharmaceutical manipulation of HAT and HDAC modulate tolerance development, OIH, the perception of pain, and addiction, it must be noted that treatment with inhibitors of HAT and HDAC can have global effects and result in epigenetic changes in the promoters and enhancers of many other genes, in addition to the investigated candidate genes. Therefore, there is a need to comprehensively study the histone acetylation changes induced by opioid treatment and the alterations induced by the use of pharmaceutical inhibitors of HATs and HDACs.

### 4.4. Histone lysine crotonylation

Histone lysine crotonylation (HKcr) is a dynamic, evolutionarily conserved acyl modification that is regulated by the action of acylases, which add the modification, and deacylases, which remove the modification (Tan et al., 2011). The well-established HAT, p300/CREB binding protein, performs crotonyl transferase activity, and histone crotonylation levels are dependent on the intracellular levels of crotonyl-CoA (Sabari et al., 2015). Class I histone deacetylases, HDAC1–3, and Sirtuin 3, were identified to remove histone crotonylation (Bao et al., 2014; Wei et al., 2017). HKcr, which is primarily observed in active gene promotors and enhancers, is linked to gene activation, and changes in HKcr were observed in various disease states (Sabari et al., 2015; Wang et al., 2021). Changes in histone lysine crotonylation were implicated in acute kidney injury (Ruiz-Andres et al., 2016), DNA damage response, and stress-induced depressive-like behaviors (Liu et al., 2019). The induction of crotonylation at the LTA of HIV is known to reactivate latent HIV (Jiang et al., 2018). Histone crotonylation was identified to be involved in neuropathic pain, the induction of neuroinflammation, and hyperalgesia (Zou et al., 2022). Histone lysine 18 crotonylation (HK18cr) is an abundant mark in the intestine, and the gut microbiota was shown to regulate histone crotonylation levels in the gut via SCFAs. Depletion of the microbiome and the consequent reduction in SCFA, especially butyrate, was shown to lead to a decrease in crotonylation (Fellows et al., 2018). Since morphine treatment was shown to modulate SCFA production (Cruz-Lebrón et al., 2021), microbiome-mediated changes in HKCr are to be expected with opioid use. However, to date, no studies have explored the changes in histone lysine crotonylation mediated by opioids.

### 4.5. Histone lysine methylation

Histone methylation involves the addition of mono-, di-, and tri-methylation to lysine and arginine residues at the N-terminals of histones. These modifications serve as binding sites for the proteins. The reader proteins, which specifically recognize the different histone-lysine modifications, are responsible for the downstream events. Histone lysine methylation is catalyzed by SET domain-containing histone lysine methyltransferases, while enzymes containing the Jumonji domain catalyze the demethylation of histone lysine residues. Methylation of H3K9 and H3K27 is linked to heterochromatin formation and transcriptional silencing, resulting in gene repression. H3K4 methylation and H3K36 methylation are generally associated with active gene expression (Lennartsson and Ekwall, 2009; Bannister and Kouzarides, 2011; MolinaSerrano et al., 2019).

Few studies have explored the role of lysine methylation in modulating morphine-induced behavioral changes. The repressive histone mark H3K9me2 was studied for its role in modulating behavioral outcomes in response to chronic morphine treatment. Repeated morphine treatment decreases G9a, a core component of the histone lysine methyltransferase that primarily catalyzes the addition of H3K9me2 in the NAc. Reduced G9a is involved in the morphine reward pathway by affecting histone methylation at *fosB*, glutamate receptors, and repetitive elements. Interestingly, overexpression of G9a reduced the rewarding response to the drug and withdrawal symptoms (Sun et al., 2012). Similar changes were also observed with cocaine self-administration and stress (Maze et al., 2010). In addition, the expression of the methyl binding protein (MeCP2) in the central nucleus of the amygdala is altered by morphine and inflammatory pain. Morphine-induced MeCp2 binds and represses the activity of G9a, contributing to morphine's rewarding behavior (Zhang et al., 2014).

While the role of the microbiome was not assessed in the abovementioned studies, the microbiome and microbial metabolism are known to affect histone methylation by modulating substrates/methyl donors (choline, betaine, methionine, SAM, and SAH) and cofactors (folate, cobalamin, riboflavin, and niacin) of the one-carbon metabolism and folate cycle (Friso et al., 2017), as detailed in the earlier DNA methylation section. In support, the low availability of SAM was shown to affect histone lysine methylation (H3K4me3) at the promoters of genes involved in innate immunity and affect the survival of *Pseudomonas* infection in *Caenorhabditis elegans* (Ding et al., 2015). Methionine restriction was shown to decrease the methylation at histone H3K4me3 in the liver by altering the one-carbon metabolism, thus affecting the SAM and SAH levels in the liver (Mentch et al., 2015). Further research is needed to identify the role of the microbiome in regulating histone methylation and its influence on behavioral responses to opioids.

## 5. Conclusion and future perspectives

Compelling data published in the past decade from three independent branches of research indicate the following: (1) The gut microbiome modulates the epigenome through the production of epigenetically active metabolites and the regulating expression of epigenetic enzymes; (2) the gut microbiome has been shown to play an important role in modulating opioid functions, such as analgesia, and contribute to comorbidities associated with chronic opioid use, including behavioral outcomes such as anxiety and depression; and (3) opioid use causes persistent, stable epigenetic alterations that change gene expression and behavioral outcomes. In this review, we have described the possible ways in which opioid-induced microbial dysbiosis, through the production of epigenetically active metabolites, may influence the epigenetic changes observed with morphine. Currently, direct evidence linking morphine-induced changes in the microbiome and microbial metabolism to the production of epigenetically active metabolites and subsequent epigenetic changes responsible for altered behavioral responses to opioids is sparse. In addition, studies aimed at understanding the role of the microbiome in mediating the epigenetic changes associated with opioid use have heavily relied on the use of pharmacological inhibitors of epigenetic enzymes, supplementation of SCFAs, and strategies based on candidate genes. To fully understand the crosstalk between the microbiome and epigenetics in modulating the pathophysiology, molecular, and behavioral changes associated with morphine use, it will be necessary to comprehend opioid and opioid microbiome-induced changes in metabolites, including choline, methionine, SAM, SAH, folate, SCFAs, and vitamins, which serve as cofactors in one-carbon metabolism and the folate cycle, by sampling the serum and fecal samples in gnotobiotic and germ-free mice.

Future studies should investigate epigenetic modifications (DNA methylation, histone acetylation, crotonylation, and methylation) in combination with the altered microbiome and epigenetically active metabolites to obtain a better understanding of the role of the microbiome in modulating opioid-induced changes in the epigenome.

## Author contributions

UK drafted the manuscript. SR provided critical revisions, approved the manuscript, and provided funding and resources. All authors contributed to the article and approved the submitted version.
